# A Systematic Review of the Psychometric Quality of Instruments for Assessing Adverse Childhood Experiences

**DOI:** 10.3390/children13081045

**Published:** 2026-08-05

**Authors:** Laura Carreres Sanchis, María Inmaculada Colorado Lluch, Manuel Martí-Vilar, Francisco González-Sala

**Affiliations:** 1 Faculty of Psychology and Speech Therapy, University of Valencia, 46010 Valencia, Spain; laucasa8@alumni.uv.es; 2 Department of Methodology of Behavioral Sciences, University of Valencia, 46010 Valencia, Spain; m.inmaculada.colorado@uv.es; 3 Department of Basic Psychology, University of Valencia, 46010 Valencia, Spain; manuel.marti-vilar@uv.es; 4 Department of Developmental and Educational Psychology, University of Valencia, 46010 Valencia, Spain

**Keywords:** psychometric properties, adverse childhood experiences, child abuse, assessment instruments, measurement quality, COSMIN, systematic review

## Abstract

**Highlights:**

**What are the main findings?**

- Various questionnaires can be used to assess adverse childhood experiences.
- Questionnaires that assess ACEs generally exhibit good psychometric properties.

**What are the implications of the main findings?**

- Cross-cultural evaluations of ACE assessment tools are required.
- Adaptations are needed for instruments that assess ACEs in populations with specific characteristics.

**Abstract:**

**Background/Objectives**: The study of adverse childhood experiences (ACEs) has become increasingly important due to its impact on the physical and mental health throughout life. The aim of this study is to assess the psychometric quality of the instruments used to measure ACEs, following the COSMIN guidelines for reviewing patient-reported outcome measures. **Methods**: A PRISMA-adherent systematic search of scientific databases was conducted to identify empirical studies analysing the psychometric properties of ACE questionnaires used with children, adolescents, and adults. Ultimately, 20 studies were included, covering various instruments such as the ACE-10, ACE-Q, ACE-SQ, ACE-IQ, SC-ACE-IQ, ACE-IQ-10 and the ACE-THL. **Results**: The results show heterogeneous psychometric evidence. Whilst properties such as internal consistency generally show adequate results, other dimensions, such as responsiveness or measurement error, are scarcely evaluated. Furthermore, methodological limitations were identified, such as the predominant use of young female samples and the lack of longitudinal studies. **Conclusions**: In conclusion, most available ACE instruments demonstrate good psychometric properties, with the ACE-IQ being the one with the strongest evidence supporting these properties across different contexts.

## 1. Introduction

Adverse childhood experiences (ACEs; hereinafter) are potentially traumatic events, such as abuse or neglect, which constitute one of the main sources of stress in childhood [1]. Because of their impact on public health and human development, the study of ACEs has become increasingly important. In healthcare, ACEs are used as an epidemiological concept to predict the risk of physical and psychological illness in adulthood. Beyond their impact on health, ACEs also carry legal implications, as legal frameworks in this area aim to establish the criminal liability of caregivers.

Child maltreatment encompasses any form of physical, emotional or sexual abuse, neglect or exploitation that may compromise a child’s health, development or dignity, particularly in contexts where protection and care should be provided, such as within the family or other positions of trust [1]. Research on this topic began with the study by Felitti and colleagues, who established a link between negative experiences in childhood and health in adulthood. This model includes three types of abuse—physical, sexual and psychological—two forms of neglect—physical and emotional—and several categories of family dysfunction, such as domestic violence, substance misuse or parental separation.

Scientific evidence has shown that ACEs are consistently associated with physical, emotional and behavioural health problems in adulthood, making early adversity a key determinant of long-term health [2,3,4]. Indeed, as noted in [2], a higher number of ACEs, combined with other risk factors, was identified as one of the main causes of mortality. Subsequent reviews have linked ACEs to an increased risk of non-communicable diseases, mental health disorders such as depression or anxiety, self-harm and substance use, and a high health and social burden [5,6,7,8], which reinforces the need for their early detection and the implementation of preventive strategies [4,9,10,11].

From an epidemiological perspective, childhood experiences have also been linked to an increased risk of premature mortality through their impact on development, risk behaviours and the early onset of chronic diseases [3]. Exposure to such adversity is associated with early-onset chronic stress, alterations in brain development and difficulties with emotional regulation, which increase vulnerability to mental health disorders, particularly among young people [4].

The concept of ACEs has evolved over time. Whilst the original classification facilitated its measurement, there is now a need to broaden its definition to include other forms of childhood adversity and to better understand its impact on development [12,13]. At the same time, some perspectives suggest that not all negative experiences should be classified as adverse, given that they differ in intensity and consequences [14]. In recent years, the concept has been expanded to include contextual factors such as poverty, discrimination or community violence, adopting an ecological perspective that recognises the influence of the social and cultural environment [4,15,16]. It also incorporates protective factors such as emotional support or the presence of secure attachment figures, which can offset the negative effects of such experiences [17,18].

### 1.1. Instruments for Evaluating ACEs

Regarding the assessment, the ACE-Q questionnaire is one of the most widely used tools for identifying exposure to ACEs through retrospective recall in adulthood. Also known as the ACE-10 or ACE-SQ, it assesses direct abuse (psychological, physical and sexual) and dysfunction within the family environment, including physical and psychological neglect, mental health problems or substance use within the family, domestic violence and parental separation [19]. This instrument provides a cumulative score, demonstrating that exposure to multiple categories of adversity is associated with an increased risk of mental health and behavioural problems.

Subsequently, the ACE-IQ questionnaire was developed with the aim of making it applicable at an international level, expanding the number of adversities assessed and incorporating dimensions such as violence outside the family environment [1]. This instrument uses a cumulative scoring system that facilitates the overall assessment of childhood adversity. Comprising 31 items, it assesses 13 categories grouped into four main dimensions: abuse, neglect, family dysfunction, and violence outside the family environment, such as bullying at school. Based on this, the abbreviated version, the ACE-IQ-10, was developed for clinical settings recognising, that even isolated experiences can significantly affect development. Cultural adaptations have also been created, such as the SC-ACE-IQ and the ACE-TH L, which incorporate socioeconomic factors and positive elements of emotional support to broaden our understanding of childhood experiences.

### 1.2. Current Study

Since Fellitti and Kaiser introduced the term ‘Adverse Childhood Experiences’ in 1998, research in this field has grown steadily, focusing primarily on the impact these experiences have on people mental health. Over time, the concept has evolved to include other experiences that were not initially considered, leading to the development of new assessment instruments. The variability among these tools and the diversity of the populations studied call for a rigorous evaluation of how well they perform. Yet, a review of the literature across various databases and in Prospero revealed a lack of studies comprehensively examining the psychometric properties of these instruments; this gap motivated the present review. Assessing these properties makes it possible to verify the validity and reliability of the questionnaires. This study conducts a systematic review in accordance with the PRISMA guidelines [20], focusing on the psychometric properties of instruments that measure adverse childhood experiences, using the COSMIN checklist [21] to ensure a rigorous evaluation of patient-reported outcomes (PROMs).

## 2. Materials and Methods

### 2.1. Search Strategy

A systematic review of the scientific literature was conducted in a structured manner, following the guidelines proposed by PRISMA [20].

The search strategy was carried out between November and December 2025 in three databases—Web of Science (Core Collection) (27 November 2025), Scopus (2 December 2025) and ProQuest (3 December 2025)—and it was conducted in three main phases with the aim of identifying as many ACE assessment instruments as possible and to minimise bias. The first phase consisted of an initial exploratory search to review the literature on ACE and identify measurement instruments. In the second phase, a systematic search was carried out in these databases, using Boolean operators to identify the largest number of articles relevant to the objective of this study. The combination of Boolean operators is shown in Table 1. The search strategy developed for each of the databases is available as a Supplementary Material Table S1 to this article.

In addition, a manual search was carried out using the selected articles themselves in order to identify any further studies. However, the review did not yield any new records.

### 2.2. Study Selection

This review included empirical studies that evaluated the psychometric properties of ACE instruments, which were published within the last 15 years and which were conducted with clinical or non-clinical samples of children, adolescents, or adults.

This age range was selected because the questionnaires assess childhood experiences retrospectively. Only scientific articles written in English or Spanish were included. Studies that did not specifically address ACE or that analysed only correlations without focusing on psychometric properties were excluded. Studies lacking empirical data and comparisons with other trauma instruments were also excluded.

### 2.3. Procedure

**Figure 1:**
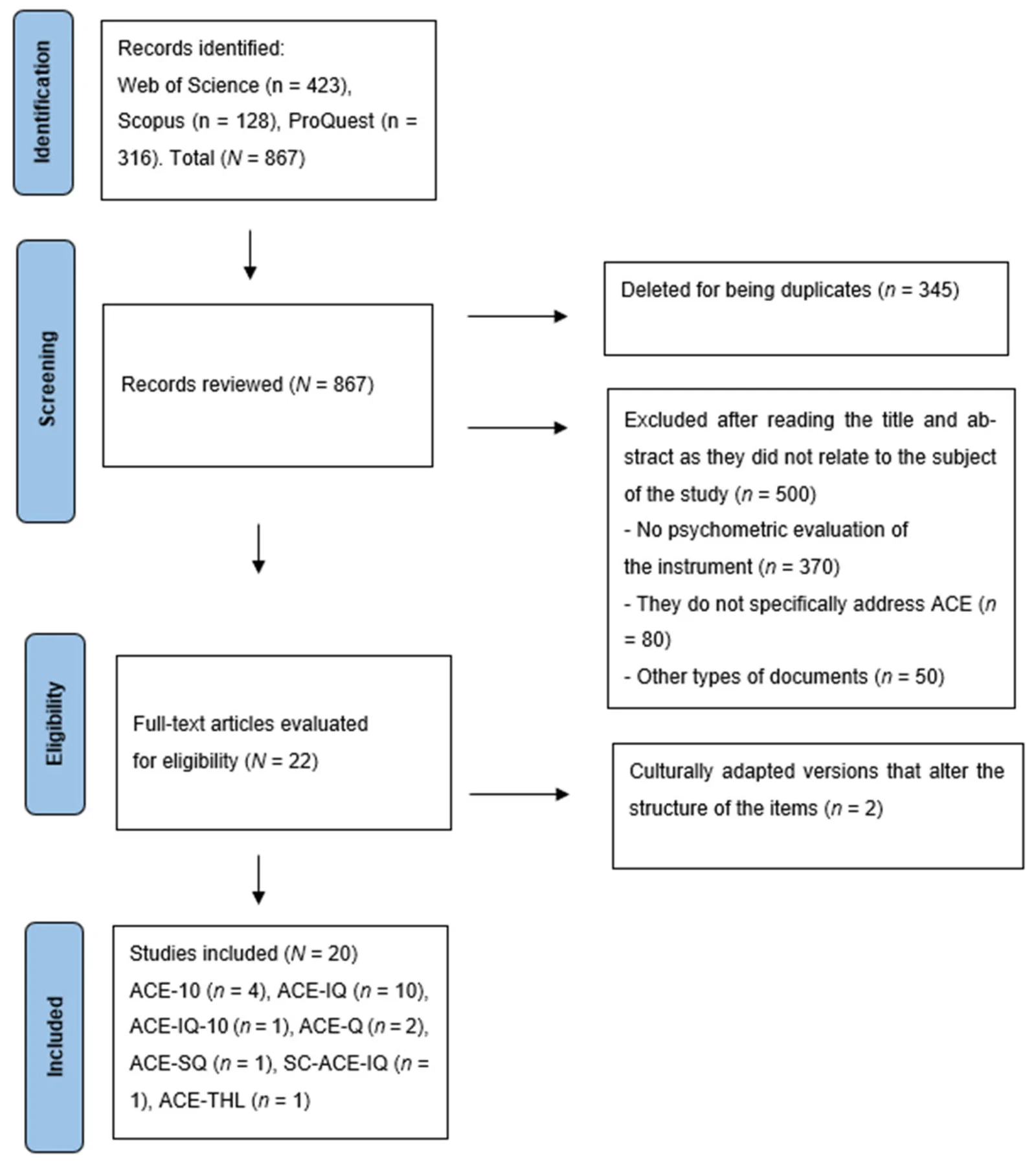
Flow of information through the different phases of a systematic review.

The systematic review was conducted using filters for document type and time period, and it was structured in four phases: identification, assessment, eligibility and inclusion. During the identification phase, 867 articles were identified: 423 from Web of Science, 128 from Scopus and 316 from ProQuest. These articles were exported to Excel to analyse the titles and abstracts, manually comparing them and identifying and removing duplicate records, thereby reducing the sample to 522 studies. Following the screening, 22 articles were selected for full review, of which two were excluded for using modified versions of the instrument. Finally, 20 studies were included and evaluated by compiling information on the name of the instrument, samples, context, methodological quality and measurement properties. All of this is summarised in the flowchart (Figure 1). All records were screened by two authors in a double-blind manner (FG-S and LC) and when there was disagreement, a third reviewer intervened (MI-C).

### 2.4. COSMIN Checklist for Systematic Reviews of PROMs

In order to rigorously assess the methodological quality of the studies included in the review, the COSMIN (Consensus-based Standards for the Selection of Health Measurement Instruments) checklist was used. This checklist is designed for systematic reviews of self-reported measurement instruments (PROMs) [21] and allows for the systematic evaluation of two essential aspects: the standards, which refer to design and implementation requirements and therefore assess methodological strength, and the criteria, which examines the instrument’s psychometric properties, including dimensions such as validity, reliability, internal consistency and discriminant validity.

Data on the psychometric properties reported in the analysed articles were collated by two researchers (F.G-S. and L.C.); in cases of disagreement, a third reviewer intervened (M.I.-C.). This information was transferred to the results tables, which summarised the characteristics of the samples (see Table 2) and the psychometric properties listed in the COSMIN checklist (see Table 3), namely: structural validity, internal consistency, cross-cultural validity/measurement invariance, reliability, measurement error, criterion validity, hypothesis testing for construct validity and responsiveness. All items comprising the checklist were rated on a 5-point scale (excellent, good, fair, poor and unknown/NA). The overall score for each box was based on the lowest score (1–5) for an item within that box [21,22]. When assessing the quality of each instrument, the GRADE criteria were followed, with each property rated on a three-point scale: sufficient (+), uncertain (?) and insufficient (−) (see Table 4), as specified in the COSMIN manual [21] and in the guidelines provided by Prinsen et al. [23] and Terwee et al. [24]. Finally, the quality of evidence for each instrument was summarised in a table (see Table 5), as suggested by Prinsen et al. [22], distinguishing between high quality evidence when several articles demonstrated good methodology or one article was of excellent quality; moderate quality evidence when there were several articles with acceptable methodology or one of good quality; limited quality evidence when the quality was acceptable; and conflicting evidence when the articles were of low quality. In cases where studies lacked information on psychometric properties, the evidence was classified as conflicting.

The COSMIN Risk of Bias checklist was applied separately for each measurement property evaluated in every included study (structural validity, internal consistency, cross-cultural validity, reliability, measurement error, criterion validity, hypothesis testing and responsiveness). Each property was rated independently according to the COSMIN manual. Following COSMIN recommendations, the methodological quality for each property was determined using the ‘worst score counts’ principle. Two reviewers (F.G.-S. and L.C.) independently performed the assessment and disagreements were resolved by discussion until consensus was reached. When consensus could not be achieved, a third reviewer made the final decision.

The main objective of using this tool was to ensure that the selection and evaluation of the instruments were carried out objectively and in a reproducible manner, thereby enabling comparison between different studies.

## 3. Results

Table 2 summarises the main characteristics of the samples used in the 20 studies included in this review, together with the administration procedure employed. The most relevant findings from these studies are presented below.

### 3.1. Characteristics of the Samples from the Studies Analysed

The 20 studies included in the review are grouped according to the instrument used: original versions, ACE-10, ACE-Q, ACE-SQ, the WHO international version, ACE-IQ and specific adaptations, ACE-IQ-10, SC-ACE-IQ and ACE-THL. Overall, the samples comprise a total of approximately 16,000 participants, mainly from the United States, Hungary, Croatia, Mexico, Chile, Italy, Russia, China, Finland, France and Malawi.

#### 3.1.1. General Overview of the Samples

1. Original versions of the ACE (ACE-10, ACE-Q, ACE-SQ).

Seven studies [25,26,27,28,40,41,42] used these classic versions with a total of 1978 participants, including both clinical populations and students and young adults from the general population. Clinical samples tend to be small (20–75 participants) and focus on contexts of social vulnerability, low socioeconomic status or residential treatment, whilst university samples are larger, with up to 792 participants. In terms of gender, female participation is predominant, with percentages ranging from 56% to 85%. Administration procedures varied, including online surveys, self-administered questionnaires and supervised interviews, but all included the 10 classic items on abuse, neglect and family dysfunction.

2. ACE-IQ International Version.

Ten studies [29,30,31,32,33,34,35,36,37,38] used the full 13-category version, with a total sample size across all articles exceeding 14,000 participants. The samples comprised adolescents and adults recruited from both general and clinical settings, with broad geographical representation across the Americas, Europe, Asia and Africa. Notable examples include large samples of school pupils and university students, such as the studies in Mexico [31] and the United States [30]. The questionnaire was administered using several methods, from online surveys to face-to-face interviews. In terms of gender and age, the sample is heterogeneous, although female participants and younger age groups predominate.

3. Specific adapted versions.

Three studies used modified versions: the SC-ACE-IQ in mainland China (12 categories) [43], the ACE-IQ-10 in the Netherlands (10 items) [39] and the ACE-THL in Finland (14 items, including protective factors) [44]. These adaptations reflect the need for cultural and methodological adjustments, as well as to incorporate protective factors and positive experiences, which broadens the perspective on childhood adversity.

#### 3.1.2. Patterns and Trends Observed

In terms of the participants’ age or developmental stage, the population is predomi-nantly made up of adolescents and university students aged between 12 and 24, followed by young adults and middle-aged adults up to the age of 50, this implies that the over-representation of young people may limit the generalizability of the results to the general adult population.

Female participation is predominant in almost all studies, particularly in clinical and university settings, suggesting a possible selection bias or a greater willingness among women to participate in this type of research.

Furthermore, the studies cover a wide range of geographical and cultural diversity, including samples from countries in North and Latin America, Europe, Asia and Africa, which demonstrates the global applicability of the instruments. However, in many cases, cultural adaptations—such as translations—were necessary to ensure that the measures were valid across different contexts.

In terms of sample type, clinical populations tend to be smaller and more specific, focusing on social vulnerability, mental health or residential treatment. In contrast general and university samples are larger, which facilitates comparisons between groups and longitudinal follow-ups.

Finally, regarding administration methods, the questionnaires were administered in various ways, including supervised interviews, paper-based self-reports and online sur-veys, which provided a degree of methodological flexibility but also led to some heterogeneity in the interpretation of the results.

### 3.2. Methodological Quality and Measurement of the Instruments Using the COSMIN Checklist

With respect to structural validity, clear differences are observed across the studies analysed. The studies that report a ‘Very Adequate’ level for the ACE-IQ instrument include Santelices et al. [29], Gette et al. [30], Casas-Muñoz et al. [31] and Muzi et al. [36], while Van der Feltz-Cornelis and De Breus et al. [36] used the ACE-1Q-10. These studies stand out for their use of confirmatory factor analysis (CFA), a robust method that tests whether the theoretical structure proposed by the instrument is supported by the data, thereby verifying whether the items adequately measure the intended dimensions.

In contrast, studies such as that by Kovács-Tóth et al. [27] using the ACE-10 are less rigorous, since they rely on an exploratory technique that is less demanding than CFA. For their part, authors such as Murphy et al. [25] and Oláh et al. [28] present ‘Undetermined’ results, reflecting a lack of sufficient information to validate the questionnaires in this respect.

In terms of internal consistency, most studies demonstrate a robust relationship between their items. The studies rated as ‘Very Adequate’ according to this criterion include Murphy et al. [25], who used the ACE-10; Casas-Muñoz et al. [31], Ho et al. [34], Tarquino Camille et al. [35], Kibitov et al. [37], Téllez et al. [38], Van der Feltz-Cornelis and De Beurs [39], who used the ACE-IQ-10 and Hietamäki et al. [44] who used the ACE-THL. However, there is one exception: the study by Gette et al. [30], which is rated as “Inadequate” for the ACE-IQ, as it obtained low internal consistency scores.

Cross-cultural validity has been assessed in a few studies. The studies by Ho et al. [34], Tarquino Camille et al. [35] and Tellez et al. [38] on the ACE-IQ, together with that by Chen et al. [43] on the SC-ACE-IQ, achieve the ‘Very Adequate’ rating by demonstrating that these versions are understood in the same way across different cultures and genders.

Regarding test–retest reliability and measurement error, there is a distinction between cross-sectional and longitudinal studies. In terms of reliability, the studies by Ho et al. [34], Schauss et al. [40] using the ACE-Q, and Chen et al. [43] and Hietamäki et al. [44] achieved the highest rating, having demonstrated the stability of responses over time. In contrast, 14 of the 20 studies provide no evidence concerning this property. In terms of measurement error, only Tarquino Camille et al. [35] and Hietamäki et al. [44] are classified as ‘Very Adequate’, whilst others such as Oláh et al. [28], Gette et al. [30] and Christoforou and Ferreira et al. [33] are rated as ‘Doubtful’, which prevents a precise determination of the magnitude of error in the questionnaire scores.

In terms of criterion validity, the studies by Santelices et al. [29], Christoforou and Ferreira et al. [33] and Van der Feltz-Cornelis and De Beurs et al. [39] stand out for their methodological rigour; however, most of the remaining authors [26,30,31,34,35,36,37,38,40,41,42] do not assess this property, which indicates a lack of evidence.

Regarding construct validity through hypothesis testing, the studies by Murphy et al. [25], Perković et al. [26], Santelices et al. [26] and Christoforou and Ferreira et al. [33] achieve the ‘Very Adequate’ level by linking ACE to other mental health disorders. On the other hand, studies such as those by Gette et al. [30] and Michael et al. [41] are rated as ‘Undetermined’.

Finally, responsiveness is the dimension for which there is the greatest lack of evidence in the sample of studies analysed. Nineteen studies classified it as ‘Not Applicable’, which implies that they did not assess the questionnaire’s sensitivity in detecting changes following treatment. The only author to present an ‘Adequate’ methodological design is Schauss et al. [40], who used the ACE-Q to conduct a nine-week follow-up, providing evidence on the questionnaire’s usefulness for monitoring clinical progression. All this data can be found in Table 3.

### 3.3. Measurement Criteria: Quality of Instruments

As shown in Table 4, structural validity and internal consistency are the properties most frequently assessed in the studies analysed, with 15 studies demonstrating adequate psychometric robustness for each of these properties.

Cross-cultural validity has been primarily assessed for the ACE-IQ instrument [29,31,32,34,35,36,37,38,39]. In contrast, the least frequently assessed psychometric properties have been reliability and responsiveness. Reliability has been assessed in only six studies [34,35,40,42,43,44], with no study assessing it for the ACE-10 instrument, while responsiveness has been assessed even less often, appearing only in the study by Schauss et al. [40].

Notable among the studies assessing a greater number of psychometric properties are those on the ACE-IQ [32,34,35], the study by Hietamäki et al. [44] on the ACE-THL instrument, and the study by Chen et al. [43] on the SC-ACE-IQ instrument.

### 3.4. Strength of Evidence

An analysis of the psychometric properties based on the criteria in the COSMIN checklist, taking into account the modified GRADE criteria [22] (see Table 5), reveals that none of the 20 studies provided information for all eight criteria assessed, although the studies providing the strongest evidence were those relating to the ACE-IQ [25,34], the ACE-IQ-10 [39] and the ACE-THL [44]. With respect to the property assessed, there is strong and moderate evidence in 75% of the studies analysed concerning structural validity, rising to 90% in relation to internal consistency.

## 4. Discussion

The aim of this study was to assess the psychometric quality of the instruments used to measure ACEs by applying the COSMIN guide [21]. Regarding the strength of the psychometric evidence for the instruments used to assess adverse childhood experiences (ACEs), considerable heterogeneity is observed. Evidence is robust for certain properties—such as internal consistency, which exceeds acceptability thresholds, and structural validity as assessed by confirmatory factor analysis (CFA)—whereas it is limited or unknown for others, such as responsiveness and measurement error.

The evidence shows that both the original 10-item versions (ACE-10, ACE-Q, ACE-SQ), the international versions (ACE-IQ) and other adaptations (ACE-IQ-10, ACE-THL) are reliable tools for assessing ACEs in children, adolescents and adults populations.

An analysis of the instruments derived from the original ACE questionnaire (ACE-10, ACE-Q and ACE-SQ) shows that there is limited robust evidence, with moderate or unknown levels predominating for several of the COSMIN properties assessed [21]. This demonstrates that, although these instruments have been widely used in the past, the psychometric evidence is limited.

Moreover, the transition from the original questionnaire to the international version has enabled the identification of other clinically relevant dimensions. The ACE-IQ is the instrument that has been evaluated in the greatest number of studies [29,30,31,32,33,34,35,36,37,38], providing a wealth of information on its psychometric properties. However, the results show varying levels of evidence: solid support is available for some properties, such as internal consistency [31,33,34,35,37,38], whereas for others, such as responsiveness, the evidence is unknown across all studies. Although this may be explained by the fact that the instruments used to assess ACEs were designed to measure exposure to such experiences rather than to track longitudinal changes.

Of the instruments analysed, the ACE-IQ and the ACE-THL are those with the most robust evidence, with 75% of the evidence being strong or moderate. However, it is worth nothing that the evidence for the ACE-THL comes from a single study by Hietamäki et al. [44]. By contrast, the ACE-IQ has a broader empirical basis [30,31,32,33,34,35,36,37,38].

Overall, the results show that internal consistency is one of the psychometric properties with the strongest evidence and empirical support. In the case of adverse childhood experiences, the use of Cronbach’s alpha or factor analysis may be inappropriate under a formative model, in which the indicators define the construct, as opposed to a reflective model, in which adversity is the cause of the indicators captured in the instrument’s items. Applying these methods under a formative model can generate erroneous estimates, since the indicators that make up the construct do not necessarily have to be interrelated [45]. For example, having experienced school-related difficulties does not necessarily have to be linked to having suffered abuse or neglect at the hands of primary careers. In such cases, as Cruz-Avelar et al. [46] point out, it is important to focus on construct validity through scientific evidence when selecting the items for the instrument. This selection should take into account the specific characteristics of the population to be assessed and the purpose of the instrument.

Other dimensions—particularly responsiveness and measurement error—have been poorly evaluated, which would explain the ‘unknown’ evidence in these areas. Measurement error, specifically, was only assessed in 13 of the 20 studies analysed. This may be related to memory biases. Since ACE instruments assess experiences retrospectively; the presence or absence of an event may therefore be affected if the individual denies or downplays it. Other biases associated with self-reports, gender, and social or cultural groups may also affect the accuracy of this measurement. Addressing these biases would require assessing whether each item within an instrument behaves in the same way across different groups. For example, certain behaviours, such as the use of violence or physical punishment in child-rearing, or abandonment and neglect, may be normalised differently depending on whether the child is a boy or a girl. All these factors also complicate the assessment of criterion validity. A further issue is that these instruments typically provide a total score based on the number of incidents the individual has experienced, without weighting the score according to the severity of the incident, the age at which it occurred, or whether the incident or incidents became chronic. However, some retrospective epidemiological studies [47,48] highlight the usefulness of this type of instrument despite the difficulties associated with memory biases.

As mentioned above, cultural biases can, to a certain extent, influence cross-cultural validity. Using these instruments across different cultures involves more than simply translating the items: concepts such as ‘abuse’, ‘mistreatment’ or ‘neglect’ are culturally defined, and a practice considered normal in one context may be regarded as a form of mistreatment in another. Existing values and prejudices towards certain communities, minorities or specific social groups, as well as the laws and customs specific to each community, make it difficult to draw comparisons between cultures and, consequently, to study cross-cultural validity.

These findings suggest a need for studies that comprehensively evaluate the psychometric properties of these instruments.

Taking into account the samples and the implementation procedures, the body of studies analysed reflects diversity in terms of the population, sample size and the procedures used in their implementation, which enriches the analysis of these adverse childhood experiences. However, this heterogeneity also entails limitations due to potential bias arising from an over-representation of females and younger age groups, which makes it difficult to compare results across studies using different instruments or cultural adaptations.

### 4.1. Limitations of the Studies Analysed

Some weaknesses identified in the review should be considered when interpreting the results. Firstly, there is heterogeneity in the psychometric quality of the assessed instruments, with a predominance of moderate or unknown results across several properties according to the COSMIN criteria [21], as mentioned above. This variability makes direct comparison between studies difficult and limits the generalizability of the conclusions.

Secondly, a large proportion of the studies include samples that are not representative of the general population, characterised by a higher proportion of young women, mainly university students. This selection bias restricts generalisation to other population groups, such as men, older people or groups in more vulnerable situations, since the magnitude of the effect observed in these samples does not necessarily reflect what would occur in the general or clinical population, thereby failing to control for other variables that may influence the results. The lack of studies that have adapted ACE questionnaires for vulnerable groups highlights a real need, particularly given that these groups (children with disabilities, neurodevelopmental disorders or those undergoing long-term hospitalisation…) may be at greater risk of experiencing adverse events. In this regard, experts should develop adaptations using clear, accessible language tailored to these individuals’ needs, validating them through interviews and pilot studies.

Furthermore, although some instruments have been translated into different languages to adapt them to different cultural contexts, the available evidence does not allow us to conclude with certainty that cultural adaptation has been carried out in all cases. Differences in social norms, experiences of adversity and, above all, the willingness to disclose certain experiences may influence the validity of the measures used, which represents a significant limitation in international studies.

Finally, the retrospective nature of most of the instruments used must be taken into account, as this may lead to recall bias, particularly in adult populations. This aspect may affect the accuracy of the information collected and, consequently, the validity of the results obtained.

Among the review’s strengths is the rigorous COSMIN methodology [21], which enables a comprehensive assessment of the psychometric properties of ACE instruments, allowing their strengths and weaknesses to be identified and serving as a guide for professionals who need to select tools for assessing ACEs. Additionally, the inclusion of recent adaptations such as the ACE-THL and the ACE-IQ-10 indicates improvements in methodology, as they incorporate protective factors, offering a broader view of ACEs. It should be noted that the ACE-IQ-10, the SC-ACE-IQ and the ACE-THL have each been evaluated in only one study [39,43,44].

Overall, the evidence demonstrates the usefulness and reliability of ACE assessment instruments, although in some respects they need to be strengthened, particularly regarding less explored properties and through longitudinal studies. This highlights the need for future research to validate the instruments more comprehensively and ensure their applicability in different contexts.

### 4.2. Limitations and Future Lines of Research

Among the limitations of this review, it is worth noting the reduced number of studies assessing the psychometric properties of some of the instruments included in this review. This may be due to the restricted number of databases used, the search strategy employed and the decision to include only articles published within the last 15 years. As a result, studies that did not include terms related to psychometric properties in their title, abstract or keywords, or that were published before the year 2000 may have been excluded. Another limitation of this review is that content validity has not been assessed. This measurement property is one of the most important in patient-reported outcome measures (PROMs) when determining which instruments exhibit the best psychometric properties [49] and it has an impact on clinical practice [50]. In the case of ACEs specifically, it is important to consider how the concept has evolved, expanding beyond the classic events to include other contextual and cultural factors, as well as protective factors [4,15,16]. This conceptual evolution has direct implications for how content validity should be assessed in ACE instruments, since items may need to reflect this broader, updated understanding of the construct. This assessment was not possible in the present review, as not all the studies analysed provided details of how the instrument had been developed. Another limitation relates to the fact that the review was not registered with PROSPERO, although prior to its commencement, a check was carried out to ensure that there were no similar reviews in the included databases or in PROSPERO itself. Among the instruments analysed in this review, it should be noted that the ACE-IQ, the SC-ACE-IQ and the ACE-THL are the ones that include the greatest number of items, some addressing stressors and others protective factors.

Regarding future lines of research, the results obtained not only highlight the need to use more comprehensive instruments, such as the international questionnaire, but also the urgency of focusing on populations that are under-represented in the current literature, such as at-risk groups, for example children in the child protection system. Furthermore, given the predominantly female samples, future research should aim to include a more balanced representation of men, as well as broaden the age ranges to include more adolescents and adults, in order to analyses possible differences in the experience of childhood adversity across the life course.

It is also crucial to consider groups that have been largely overlooked in the studies reviewed, such as people with disabilities. In particular, children and adolescents with disabilities and chronic illnesses face a significantly higher risk of experiencing abuse, neglect and violence, with rates exceeding 70 per cent depending on the type of violence suffered. These rates are even higher in the case of girls and adolescent women with disabilities, as highlighted in the micro-report on violence against children and adolescents with disabilities in Spain [43]. It is therefore necessary to adapt existing assessment tools to this population, thereby enabling the collection of valid and reliable data from these groups.

Moreover, although the international questionnaire has been considered suitable for use across different cultural contexts, its use alone does not guarantee adequate cultural sensitivity. Future research should explore its application in specific contexts, such as migrant or refugee populations, where exposure to collective violence plays a key role, as well as in cultures where factors such as stigma may make it difficult for individuals to disclose these adverse experiences. This implies not only linguistic adaptation but also taking into account specific cultural and contextual factors that may affect the validity of the questionnaire and the epidemiological studies themselves, thereby skewing reporting rates. Furthermore, a review of administration procedures and data collection strategies is required, particularly with regard to vulnerable groups.

Taken together, these results lead to the conclusion that the instruments analysed are suitable for assessing ACEs, given their robust psychometric properties—primarily relating to internal consistency and construct validity. However, they also suggest that progress in ACE assessment does not depend solely on developing more comprehensive instruments, but on ensuring that these tools capture the true diversity of populations and remain inclusive, culturally sensitive and accessible. This would not only improve the quality of research but also lead to a fairer and more comprehensive understanding of the impact of childhood trauma.

From a statistical and psychometric perspective, those instruments that demonstrate greater validity and reliability may be superior to those that score lower on these constructs. Furthermore, it is important to ensure that these psychometric properties are maintained across different cultures, contexts and samples. It can be concluded that, amongst the instruments analysed in this review, the World Health Organization’s ACE-IQ [1] is the one with the greatest number of studies evaluating its psychometric properties. Most of these studies demonstrate good structural validity and internal consistency, and the instrument has also been evaluated across a wider range of cultural contexts, showing good cross-cultural validity. However, it should be noted that the quality of the evidence varies according to the psychometric domain and that measurement error and responsiveness remain insufficiently established.

In light of the results obtained, the ACE-IQ may be the most suitable tool for conducting epidemiological studies; however, given the dynamic nature of adverse experiences in childhood, it would be advisable to review the instruments to improve their content validity and ensure that they are adapted to the norms, customs and traditions of each culture.

## Figures and Tables

**Table 1:** Keywords used for the Boolean search.

Questionnaires	Psychometric Properties
Adverse Childhood Experiences OR ACE Questionnaire 71	validation OR reliability OR validity OR factor analysis OR internal consistency OR measurement properties OR retest
Adverse Childhood Experiences Questionnaire OR ACE-THL 71	
Adverse Childhood Experiences Questionnaire OR ACE 81	
Adverse Childhood Experiences International Questionnaire OR ACE-IQ 9	

**Table 2:** Characteristics of the samples used in scientific articles published on the psychometric properties of questionnaires that assess adverse childhood experiences.

Study and Instrument	Application Procedure	Country. Sample Size. Sample Characteristics
Murphy et al. (2014)—ACE-10 [25]	The ACE-10 questionnaire and the Adult Attachment Interview (AAI) were completed. Duration: NS.	USA (NY). *N* = 75. Mothers aged between 19 and 50, recruited into two groups: a clinical sample (*n* = 41) and a community sample (*n* = 34). A Hispanic/African American sample, with an income of less than $20,000 and a low level of education. The community sample was predominantly Caucasian, with an income above $40,000 and a higher level of education.
Perković et al. (2025)—ACE-10 [26]	Data was collected using Survey Monkey. Duration: NS.	Croatia. *N* = 293. Young Croatians with an average age of 22 (M = 22.34, SD = 13.40), of whom 56.9% were women (*n* = 167).
Kovacs-Tóth et al. (2023)—ACE-10 [27]	The data were collected through a set of self-reports. Duration: NS.	Hungary. *N* = 792. Hungarian adolescents aged between 12 and 17 (M = 14.98) from rural and urban schools, comprising 322 boys (40.65%) and 470 girls (59.34%).
Oláh et al. (2023)—ACE-10 [28]	Anonymous questionnaire administered in group sessions supervised by a health psychologist. Duration: NS.	Hungary. *N* = 240. Hungarian adolescents aged between 12 and 17 receiving child protection services.
Santelices et al. (2025)—ACE-IQ [29]	Self-administered survey with support from a psychologist. Duration: NS.	Chile. *N* = 705. Final analysis included 651 participants with complete questionnaire data. Randomised sample stratified by sex. Participants enrolled in MAUCO (Maule Cohort of Chronic Diseases in Chile). Participants had a mean age of 48.74 years (SD = 6.34), of whom 61.1% were women.
Gette et al. (2022)—ACE-IQ [30]	Online questionnaire in exchange for credits towards their courses. The data was collected over two and a half years.	USA (TX). *N* = 5183. University students with a mean age of 19.10 years (SD = 2.56), of whom 70.3% were women. The racial identity breakdown of the sample was as follows: 58.05% white, 19.73% Hispanic/Latino, 10.13% multiracial, 6.56% African American, 4.31% Asian/Asian American, 0.40% Arab, 0.40% Native American and 0.42% other/not reported.
Casas-Muñoz et al. (2024)—ACE-IQ [31]	Participants invited through social media networks of the selected schools	Mexico. *N* = 5836. Students at state upper secondary schools in 20 states in Mexico. The age range was 11 to 19 years (M = 16.13) (SD = 1.32), of whom 38.99% were male (SD = 2.276) and 61.01% were female (SD = 3.560).
Kidman et al. (2019)—ACE-IQ [32]	The interviews were conducted in the homes of the adolescents using a tablet by an interviewer trained in the language. Duration: NS.	Malawi. *N* = 410. Adolescents aged between 10 and 16 (M = 12.99, SD = 1.74), of whom 47% were girls and 94% were in education, and their primary carers, a group comprising mainly women (91%)
Christoforou and Ferreira (2020)—ACE-IQ [33]	Online survey using Google Forms. Duration: approximately 15 min per participant.	Cyprus. *N* = 284. Adults aged between 18 and 51, with a mean age of 23.4 (SD = 5.7), of whom 77.5% were women. A key requirement was that participants had not experienced suicidal thoughts.
Ho et al. (2019)—ACE-IQ [34]	Translation of the questionnaire into traditional Chinese and administration of the questionnaire online. Duration: NS.	China. *N* = 433. Chinese adults aged between 18 and 24 from two universities in Hong Kong. The average age of the participants in this sample was 20.16 years (SD = 1.67); 178 were men (41.1%) and 218 were associate degree students (50.3%).
Tarquino Camille et al. (2023)—ACE-IQ [35]	Translation of the questionnaire into French and administration in two parts: the first part is online, anonymous, and self-administered and the second part is completed 15 days later. Duration: NS.	France. *N* = 367. 78.2% were women (*n* = 273), with a mean age of 37.1 years (SD = 14). In the initial questionnaire, the sample comprised 367 adults aged 18 and over; at the follow-up, an 88% retention rate was achieved, and the sample comprised 322 participants.
Muzi et al. (2025)—ACE-IQ [36]	Participants from the researchers’ personal circles and their participants completed the questionnaire independently. Duration: NS.	Italy. *N* = 1205. Convenience sampling was used. The average age was 40.68 years (SD = 17.57) and 630 of the participants were women.
Kibitov et al. (2024)—ACE-IQ [37]	Face-to-face administration. Duration: NS.	Russia. *N* = 123. Adults aged 18 and over, of whom 88 were women (mean age = 25) with a clinical condition (68 participants with depression and 55 without a psychiatric diagnosis).
Téllez et al. (2023)—ACE-IQ [38]	Data collection was carried out online through Google Forms, disseminated via Facebook and WhatsApp.	Mexico. *N* = 917. Adults aged 18 to 75, selected through non-probabilistic convenience sampling, of whom 79.3% were women (*n* = 727).
Van der Feltz-Cornelis & De Beurs, (2023)—ACE-IQ-10 [39]	Translation of the questionnaire into Dutch and administration. Duration: NS.	The Netherlands. Sample 1 (*n* = 298) from an outpatient mental health centre was assessed using the ACE-IQ-10 questionnaire, whilst Sample 2 (*n* = 234) from another mental health centre was administered a different questionnaire.
Schauss et al. (2021)—ACE-Q [40]	Participants completed the questionnaire during the first week at the treatment centre and were reassessed after 9 weeks.	USA. *N* = 20. Adolescents aged between 11 and 17.
Michael et al. (2025)—ACE-Q [41]	Participants completed the online questionnaire. Duration: NS.	USA. *N* = 357. University students aged between 18 and 22, of whom 63.2% were women.
Zanotti et al. (2018)—ACE-SQ [42]	Participants completed the questionnaire manually in small group sessions at two separate times over the course of a year.	USA. *N = 141*. NCAA student athletes. Median age 20 (M = 19.55, SD = 1.12), of whom 46.8% were men.
Chen et al. (2022)—SC-ACE-IQ [43]	Translation of the questionnaire into Chinese and administration of the final version online. Duration: NS.	China. *N* = 566. Health sciences students. The mean age of the participants was 22 years (SD = 2.83), of whom 74.8% were men.
Hietamäki et al. (2023)—ACE-THL [44]	Online application in two phases: the first phase involves interviews, and the second involves a test–retest application two weeks later. Duration: NS.	Finland. *N* = 20. Cognitive interviews: 50% women, average age: 59 years. Quantitative study: *N* = 513 adults in the first phase and *N* = 426 in the second.

Note: NS: not specified.

**Table 3:** Psychometric properties and methodological quality of the instruments according to the COSMIN guidelines.

Psychometric Property	Articles	Psychometric Property	Articles
***Structural validity***		***Measurement error***	
Excellent	[29,30,31,36,39]	Excellent	[35,44]
Good	[26,27,32,33,34,35,37,38,41,44]	Good	[26,31,32,34,36,37,40,42]
Fair		Fair	[28,30,33]
Poor		Poor	
Unknown/NA	[25,28,40,42,43]	Unknown/NA	[25,27,29,38,39,41,43]
***Internal consistency***		***Criterion validity***	
Excellent	[25,31,33,34,35,37,38,39,44]	Excellent	[29,33,39]
Good	[26,27,28,29,32,36,41,42,43]	Good	[25,27,28,32,43,44]
Fair		Fair	
Poor	[30]	Poor	
Unknown/NA	[40]	Unknown/NA	[26,30,31,34,35,36,37,38,40,41,42]
***Cross-cultural validity/*** ***Measurement invariance***		***Hypothesis testing for*** ***construct validity***	
Excellent	[34,35,38,43]	Excellent	[25,26,29,33]
Good	[26,27,29,31,32,36,37,39,44]	Good	[27,28,32,34,35,36,37,38,39,43]
Fair		Fair	
Poor		Poor	
Unknown/NA	[25,28,30,33,40,41,42]	Unknown/NA	[30,31,40,41,42,44]
***Reliability***		***Responsiveness***	
Excellent	[34,40,43,44]	Excellent	
Good	[35,42]	Good	[40]
Fair		Fair	
Poor		Poor	
Unknown/NA	[25,26,27,28,29,30,31,32,33,36,37,38,39,41]	Unknown/NA	[25,26,27,28,29,30,31,32,33,34,35,36,37,38,39,41,42,43,44]

Note: ACE-10: Murphy et al. (2014) [25]; Perković et al. (2025) [26]; Kovacs-Tóth set al. (2023) [27]; Oláh et al. (2023) [28]. ACE-IQ: Santelices et al. (2025) [29]; Gette et al. (2022) [30]; Casas-Muñoz et al. (2024) [31]; Kidman et al. (2019) [32]; Christoforou and Ferreira (2020) [33]; Ho et al. (2019) [34]; Tarquino Camille et al. (2023) [35]; Muzi et al. (2025) [36]; Kibitov et al. (2024) [37]; Téllez et al. (2023) [38]. ACE-IQ-10: Van der Feltz-Cornelis and De Beurs (2023) [39]. ACE-Q: Schauss et al. (2021) [40]; Michael et al. (2025) [41]. ACE-SQ: Zanotti et al. (2018) [42]. SC-ACE-IQ: Chen et al. (2022) [43]; ACE-THL: Hietamäki et al. (2023) [44].

**Table 4:** COSMIN results of the criteria of measurement (quality of the PROM).

Study and Instrument	Structural Validity	Internal Consistency	Cross-Cultural Validity Measurement Invariance	Reliability	Measurement Error	Criterion Validity	Hypothesis Testing for Construct Validity	Responsiveness
Murphy et al. (2014)—ACE-10 [25]	?	+	?	?	?	+	+	?
Perković et al. (2025)—ACE-10 [26]	+	+	+	?	+	?	+	?
Kovacs-Tóth et al. (2023)—ACE-10 [27]	+	+	+	?	?	+	+	?
Oláh et al. (2023)—ACE-10 [28]	?	+	?	?	?	+	+	?
Santelices et al. (2025)—ACE-IQ [29]	+	+	+	?	?	+	+	?
Gette et al. (2022)—ACE-IQ [30]	+	−	?	?	?	?	?	?
Casas-Muñoz et al. (2024)—ACE-IQ [31]	+	+	+	?	+	?	?	?
Kidman et al. (2019)—ACE-IQ [32]	+	+	+	?	+	+	+	?
Christoforou & Ferreira (2020)—ACE-IQ [33]	+	+	?	?	?	+	+	?
Ho et al. (2019)—ACE-IQ [34]	+	+	+	+	+	?	+	?
Tarquino Camille et al. (2023)—ACE-IQ [35]	+	+	+	+	+	?	+	?
Muzi et al. (2025)—ACE-IQ [36]	+	+	+	?	+	?	+	?
Kibitov et al. (2024)—ACE-IQ [37]	+	+	+	?	+	?	+	?
Téllez et al. (2023)—ACE-IQ [38]	+	+	+	?	?	?	+	?
Van der Feltz-Cornelis & De Beurs (2023)—ACE-IQ-10 [39]	+	+	+	?	?	+	+	?
Schauss et al. (2021)—ACE-Q [40]	?	?	?	+	+	?	?	+
Michael et al. (2025)—ACE-Q [41]	+	+	?	?	?	?	?	?
Zanotti et al. (2018)—ACE-SQ [42]	?	+	?	+	+	?	?	?
Chen et al. (2022)—SC-ACE-IQ [43]	?	+	+	+	?	+	+	?
Hietamäki et al. (2023)—ACE-THL [44]	+	+	+	+	+	+	?	?

Note: +: Sufficient; −: Insufficient; ?: Indeterminate.

**Table 5:** Strength of evidence for each study.

Instrument	Structural Validity	Internal Consistency	Cross-Cultural Validity/ Measurement Invariance	Reliability	Measurement Error	Criterion Validity	Hypothesis Testing for Construct Validity	Responsiveness	% Strong–Moderate Evidence
ACE-10	M	M	M	U	L	M	S	U	62.5%
ACE-IQ	**S**	S	S	M	L	M	M	U	75%
ACE-IQ-10	**S**	S	M	U	U	S	M	U	62.5%
ACE-Q	M	M	U	S	M	U	U	U	50%
ACE-SQ	U	M	U	M	M	U	U	U	37.5%
SC-ACE-IQ	U	M	S	S	U	M	M	U	62.5%
ACE-THL	M	S	M	S	S	M	U	U	75%
**Evidence**									
**% strong–moderate**	71.4%	100%	71.4%	85.7%	42.8%	71.4%	57.2%	0%	
**% limited conflicting**	0%	0%	0%	0%	28.6%	0%	0%	0%	
**% unknown**	28.6%	0%	28.6%	14.3%	28.6%	28.6%	42.8%	100%	

Note: S: Strong; M: Moderate; L: Limited; U: Unknown.

## Data Availability

No new data were created or analysed in this study.

## Supplementary Materials

The following supporting information can be downloaded at: https://www.mdpi.com/article/10.3390/children13081045/s1, Table S1: Keywords used for the Boolean search.
