# Supplementary material for: A Systematic Review of the Psychometric Quality of Instruments for Assessing Adverse Childhood Experiences
Source: Children (Basel). 2026 Aug 5;13(8):1045. doi: 10.3390/children13081045 (PMC13510803; doi:10.3390/children13081045)
Supplement: Supplementary file 1 [file children-13-01045-s001.zip › children-4430737-supplementary.pdf]

Table S1. Keywords used for the Boolean search.

Database	Search equation
WoS	TOPIC (Adverse Childhood Experiences OR ACE Questionnaire) AND TOPIC (validation OR reliability OR validity OR factor analysis OR internal consistency OR measurement properties OR retest)
	TOPIC (Adverse Childhood Experiences Questionnaire OR ACE-THL) AND TOPIC (validation OR reliability OR validity OR factor analysis OR internal consistency OR measurement properties OR retest)
	TOPIC (Adverse Childhood Experiences Questionnaire OR ACE) AND TOPIC (validation OR reliability OR validity OR factor analysis OR internal consistency OR measurement properties OR retest)
	TOPIC (Adverse Childhood Experiences-International Questionnaire OR ACE-IQ) AND TOPIC (validation OR reliability OR validity OR factor analysis OR internal consistency OR measurement properties OR retest)
Scopus	TITLE-ABS-KEY (Adverse Childhood Experiences OR ACE Questionnaire) AND TITLE-ABS-KEY (validation OR reliability OR validity OR factor analysis OR internal consistency OR measurement properties OR retest)
	TITLE-ABS-KEY (Adverse Childhood Experiences Questionnaire OR ACE-THL) AND TITLE-ABS-KEY (validation OR reliability OR validity OR factor analysis OR internal consistency OR measurement properties OR retest)
	TITLE-ABS-KEY (Adverse Childhood Experiences Questionnaire OR ACE) AND TITLE-ABS-KEY (validation OR reliability OR validity OR factor analysis OR internal consistency OR measurement properties OR retest)
	TITLE-ABS-KEY (Adverse Childhood Experiences-International Questionnaire OR ACE-IQ) AND TITLE-ABS-KEY (validation OR reliability OR validity OR factor analysis OR internal consistency OR measurement properties OR retest)
ProQuest	noft (Adverse Childhood Experiences OR ACE Questionnaire) AND noft (validation OR reliability OR validity OR factor analysis OR internal consistency OR measurement properties OR retest)
	noft (Adverse Childhood Experiences Questionnaire OR ACE-THL) AND noft (validation OR reliability OR validity OR factor analysis OR internal consistency OR measurement properties OR retest)
	noft (Adverse Childhood Experiences Questionnaire OR ACE) AND noft (validation OR reliability OR validity OR factor analysis OR internal consistency OR measurement properties OR retest)
	noft (Adverse Childhood Experiences-International Questionnaire OR ACE-IQ) AND noft (validation OR reliability OR validity OR factor analysis OR internal consistency OR measurement properties OR retest)
